# *HvNCX*, a prime candidate gene for the novel qualitative locus *qS7.1* associated with salinity tolerance in barley

**DOI:** 10.1007/s00122-023-04267-4

**Published:** 2023-01-19

**Authors:** Juan Zhu, Hui Zhou, Yun Fan, Yu Guo, Mengna Zhang, Sergey Shabala, Chenchen Zhao, Chao Lv, Baojian Guo, Feifei Wang, Meixue Zhou, Rugen Xu

**Affiliations:** 1 Key Laboratory of Plant Functional Genomics of the Ministry of Education / Jiangsu Key Laboratory of Crop Genomics and Molecular Breeding/ Jiangsu Co-Innovation Center for Modern Production Technology of Grain Crops/ Institutes of Agricultural Science and Technology Development, Yangzhou University, Yangzhou, 225009 China; 2 Tasmanian Institute of Agriculture, University of Tasmania, Private Bag 1375, Prospect, TAS 7250 Australia; 3 International Research Centre for Environmental Membrane Biology, Foshan University, Foshan, 528000 China; 4 School of Biological Sciences, University of Western Australia, Perth, WA 6009 Australia

## Abstract

**Key Message:**

A major QTL (qS7.1) for salinity damage score and Na+ exclusion was identified on chromosome 7H from a barley population derived from a cross between a cultivated variety and a wild accession. qS7.1 was fine-mapped to a 2.46 Mb physical interval and HvNCX encoding a sodium/calcium exchanger is most likely the candidate gene.

**Abstract:**

Soil salinity is one of the major abiotic stresses affecting crop yield. Developing salinity-tolerant varieties is critical for minimizing economic penalties caused by salinity and providing solutions for global food security. Many genes/QTL for salt tolerance have been reported in barley, but only a few of them have been cloned. In this study, a total of 163 doubled haploid lines from a cross between a cultivated barley variety Franklin and a wild barley accession TAM407227 were used to map QTL for salinity tolerance. Four significant QTL were identified for salinity damage scores. One (*qS2.1*) was located on 2H, determining 7.5% of the phenotypic variation. Two (*qS5.1* and *qS5.2*) were located on 5H, determining 5.3–11.7% of the phenotypic variation. The most significant QTL was found on 7H, explaining 27.8% of the phenotypic variation. Two QTL for Na^+^ content in leaves under salinity stress were detected on chromosomes 1H (*qNa1.1*) and 7H(*qNa7.1*). *qS7.1* was fine-mapped to a 2.46 Mb physical interval using F_4_ recombinant inbred lines. This region contains 23 high-confidence genes, with *HvNCX* which encodes a sodium/calcium exchanger being most likely the candidate gene. *HvNCX* was highly induced by salinity stress and showed a greater expression level in the sensitive parent. Multiple nucleotide substitutions and deletions/insertions in the promoter sequence of *HvNCX* were found between the two parents. cDNA sequencing of the *HvNCX* revealed that the difference between the two parents is conferred by a single Ala77/Pro77 amino acid substitution, which is located on the transmembrane domain. These findings open new prospects for improving salinity tolerance in barley by targeting a previously unexplored trait.

**Supplementary Information:**

The online version contains supplementary material available at 10.1007/s00122-023-04267-4.

## Introduction

Salinity is one of the major abiotic stresses limiting crop yield. It is estimated that nearly half of irrigated land is adversely affected by salinity (Setter and Waters 2003; Zhu 2001). Salinity imposes detrimental effects on plant growth and metabolism by imposing osmotic stress, causing ionic disbalance and increased ROS content in plant tissues (Munns and Tester 2008; Zhao et al. 2020). Identifying superior alleles for improving salinity tolerance, developing molecular markers and introducing tolerance genes into varieties by marker-assisted selection are considered the most effective approaches for successful breeding (Ribaut and Hoisington 1998).

Plants avoid the damage caused by high salt concentrations in the soil by sensing salt stress, transmitting signals to cells and adjusting cellular characteristics. The cytosolic Ca^2+^ was triggered within seconds to minutes under salt stress (van Zelm et al. 2020).

Therefore, identifying proteins or other components required for the rapid influx of Ca^2+^ under stress conditions is considered a good way to discover pressure sensors. A salt stress sensor GIPC was identified in a *moca1* mutant in *Arabidopsis* (Jiang et al. 2019). *MOCA1*, encoding a glucuronosyltransferase at the plasma membrane, plays a function in the biosynthesis of glycosyl inositol phosphorylceramide (GIPC) sphingolipids and is required for salt-induced depolarization of the cell-surface potential, Ca^2+^ spikes and waves, Na^+^/H^+^ antiporter activation. GIPCs directly bind to Na^+^ and open a Ca^2+^ channel to induce downstream responses to salinity. *moca1* mutation is defective in salt-induced Ca^2+^ spikes and SOS pathway and is hypersensitive to salt stress (Jiang et al. 2019). The Ca^2+^ sensors SOS3/CBL4 and CBL8 were identified in *Arabidopsis* and were activated by distinct Ca^2+^-signal amplitudes. Different from SOS3/CBL4-SOS2/CIPK24-SOS1 axis which confers basal salt tolerance, the CBL8-SOS2/CIPK24-SOS1 module is activated only under severe salt stress (Steinhorst et al. 2022).

The key to plant salt tolerance under a salty environment is to maintain a high cytoplasmic potassium/sodium ion ratio and intracellular ion homeostasis, which involves two mechanisms of salinity tolerance: sodium expulsion and tissue tolerance (Assaha et al. 2017). Fundamental studies in the model plant *Arabidopsis* have revealed many genes that are required for salt tolerance. The salt overly sensitive (SOS) pathway was first discovered in *Arabidopsis* using *sos1* mutants which is a typical CBL-CIPK signaling system dependent on cytoplasmic Ca^2+^(Halfter et al. 2000). Na^+^ excluding functions of SOS1 has been confirmed in many plants such as wheat (Zhu et al. 2016), rice (Martínez-Atienza et al. 2007), tuber mustard (Cheng et al. 2019), and *Populus euphratica* (Wu et al. 2007). HKT plays a key role in plant salt tolerance by controlling root to shoot Na^+^ partitioning (Munns and Tester 2008). *AtHKT1;1* loads excessive Na^+^ into the phloem and reduces excessive Na^+^ accumulation in the shoot, which mediates the long-distance transportation of Na^+^. The ortholog gene of *AtHKT1;1* in rice, *OsHKT1;5*, provides rice salinity tolerance by removing Na^+^ from the xylem sap into the surrounding xylem parenchyma cells, thereby protecting leaves from Na^+^ toxicity (Ren et al. 2005). NHXs, localized in the tonoplast membrane, are essential for Na^+^ detoxification via sequestration of Na^+^ within the vacuole under salt stress (Barragán et al. 2012). The plasma membrane H^+^-ATPase and vacuole membrane V-ATPase and PPase establish the electrochemical proton gradient across membranes to increase plant adaptation to salt (Munns et al. 2020).

Many studies identifying QTL for salinity tolerance have been performed in barley based on many morphological, physiological, and biochemical indices such as yield and agronomic traits (Xue et al. 2010), leaf chlorosis (Xu et al. 2012; Zhou et al. 2012), seed germination (Angessa et al. 2017; Witzel et al. 2010), sodium content and Na^+^/K^+^ ratio (Nguyen et al. 2013; Xue et al. 2009), ROS formation (Gill et al. 2019), and photosynthetic traits (Gill et al. 2019). Altogether, more than 70 major QTL for salinity tolerance have been identified in barley (Zhang et al. 2017). These QTL are distributed on nearly all chromosomes. Among all QTL, only *HvNax4* and *HvNax3*, which control Na^+^ exclusion, were fine-mapped. *HvNax3* was mapped to a 0.4 cM genetic interval, and *HVP10* encoding vacuolar pyrophosphatase was predicted as the candidate gene (Shavrukov et al. 2013). *HvNax4* was delimited to a 200 kb region on chromosome 1H, containing a total of 34 predicted genes with *HvCBL4,* a *SOS3* homolog gene, being suggested as the most likely candidate gene (Rivandi et al. 2011). Several genes involved in Na^+^ or K^+^ transport have been functionally characterized in barley. *HvHKT1* is most likely related to Na^+^ uniport in roots (Haro et al. 2005). *HvHKT1;5* encodes a plasma membrane protein located in root stele cells and negatively regulates salt tolerance by controlling Na^+^ unloading from the xylem and its transportation to shoots (Huang et al. 2020). Vacuolar H^+^-pyrophosphatase *HVP10* enhances salt tolerance via promoting Na^+^ translocation into root vacuoles by acting synergistically with Na^+^/H^+^ antiporters (*HvNHX1* and *HvNHX4*) to enhance H^+^ efflux and K^+^ maintenance in roots (Fu et al. 2022).

Barley (*Hordeum vulgare* L.) is the fourth largest cereal crop grown worldwide. As a result of its domestication, modern barley cultivars have become more sensitive to environmental changes and stresses; this is also true for salinity stress. Wild barley (*Hordeum spontaneum*) is the progenitor of cultivated barley and provides a rich source of genetic variations for barley improvement (Liu et al. 2020). A wild barley accession, TAM407227, showed much better salinity tolerance than cultivated barley accessions (Ma et al. 2015). Thus, it was used to construct a mapping population with a cultivated barley Franklin to identify new salt tolerance QTL/genes. From this population, we identified a major QTL for salinity tolerance and Na^+^ exclusion. Further fine mapping, along with allele sequencing, and mRNA expression analysis of a candidate gene revealed that the *HvNCX*, which encodes a sodium/calcium exchanger, was the likely gene responsible for the reported phenotype and could be targeted in breeding programs aiming to regain salinity tolerance in elite barley germplasm.

## Materials and methods

### Plant materials and genotype

A doubled haploid (DH) population consisting of 163 DH lines was produced from F_1_ of a barley cross between Franklin and TAM407227. Franklin is an Australian malting barley variety that is susceptible to salinity stress, while TAM407227 is a wild barley accession with superior tolerance to salinity (Ma et al. 2015) and is introduced from Australian Grains Genebank. The 2021 genome of v3. Morex was used as the reference genome (Index of/pub/plants/release-54/fasta/hordeum_vulgare/dna). The DH population was genotyped using DArTseq by Diversity Arrays Technology (DArT) Pty. Ltd. After deleting markers with more than 10% missing data and markers with the same scores in the population (thus the same genetic map positions), a total of 3,018 high-quality markers were used for QTL analysis (Table S1). Two DH lines differing in the major QTL on 7H but with similar agronomic traits and the same genotype of other QTL (Fig. S1) were selected for producing recombinant inbred lines (RILs). From F_2_ to F_3_, two flanking Indel markers which cover *qS7.1* region were used to select heterozygous genotypes. The F_3_ population was sown in the field for further selection of recombinant lines.

### Treatment and salinity damage score evaluation

The DH population and parents were grown in 100 cm × 160 cm × 60 cm tanks (filled with pine bark/loam-based potting mixture) under a well-designed irrigation/treatment system. 300 mM NaCl solution was used as the treatment. A control experiment was not conducted since it had been proven that different varieties, in the same potting mixture without salt, exhibited no obvious symptoms of leaf chlorosis or wilting (Zhou et al. 2012). Each genotype comprised three replicates, each of four seeds, and the experiment was arranged as a randomized complete block design. The experiments were conducted three times at the Tasmanian Institute of Agriculture, Launceston, Australia, during the consecutive growing seasons in the year 2015, 2016 and 2017. Plants were grown under a conventional glasshouse under 25/15 (± 5)°C with natural daylight cycles. The treatments started at the three-leaf stage and lasted four weeks. The application of treatment follows cycling drainage and refilling which stably maintained the targeted salt concentration in the system, similar to previously described with some modifications (Zhou et al. 2012; Fan et al. 2015, 2016). The system approached at a steady state where, after 4–5 watering cycles, NaCl additions were minimal and only water was added to compensate evaporation and transpiration. When the most susceptible lines exhibited severe symptoms, a combined score system reflecting plant damages by assessing leaf chlorosis and plant survival was used and each line was assigned damage scores from 0 to 10 (0 represents no visual effects and 10 represents all dead; scores between 0 and 5 are basically the level of leaf chlorosis and the number of dead leaves and score 6 and 10 are the percentage of plant survival as well as dead leaves and leaf chlorosis of survived plants).

For the evaluation of salinity damage scores of recombinant lines, four seeds of each recombinant line were sown in small pots (10 cm × 10 cm × 20 cm) filled with potting mixture. At three-leaf stage, 300 mM NaCl solution was added to the pots to start the treatment. The recombinants were evaluated visually based on leaf chlorosis and plant survival levels which clearly separate the genotypes into two groups: tolerant (T) and sensitive (S). The qS7.1 interval was confirmed by the genotype and phenotype of recombinant lines.

### Determination of Na^+^ content

The third fully expanded leaf from the trials under salinity stress was collected after 10 days of salt treatment in 2016 and 2017. The trial design and replications were the same as the trials for salinity damage score. Leaf sap was extracted by the freeze–thaw method (Cuin et al. 2008) and evaluated for Na^+^ content using a flame photometer (PF97, VWR International, Murarrie, Australia).

### QTL analysis and fine mapping

The linkage map (Table S2) was constructed by a software package Join Map v4.0. QTL analysis was performed using the software MapQTL6.0 followed the procedure described by Fan (Fan et al. 2015) using average values of the traits (Table S3). Two LOD support intervals around each QTL were established by taking the two positions, left and right of the peak, those have LOD values of one less than the maximum. The RIL population construction and the method of fine mapping are shown in Fig.S2. The 2021 reference genome of v3. Morex was used as a road map to narrow both genetic and physical intervals around the *qS7.1* resistance locus. Based on the barley pan-genome sequences, the deletion/insertion of genome sequencing among 20 varieties was searched by blasting on the website GrainGenes (https://wheat.pw.usda.gov/GG3/). Fragments on the two sides of the *qS7.1* interval with the base deletion/insertion of more than 8 bp between parents were converted into molecular markers. The flanking markers were screened between parents and polymorphic flanking markers of *qS7.1* were used to score the plants to identify recombinant lines. Then, additional markers in the *qS7.1* region were used to fine map the region. The *qS7.1* interval was narrowed down by comparing the genotype and the phenotype of recombinant lines.

### Sequencing and expression analysis

The primers for candidate gene sequencing and expression analysis are listed in Table S4. The amplified PCR products for sequencing analysis were separated by 1% agarose gels, and the target fragments were purified via a PureLink® Quick Gel Extraction Kit (Invitrogen, USA). All sequencing reactions were performed at the Australian Genome Research Facility (Melbourne, Australia).

The parents were sown in small pots (10 cm × 10 cm × 20 cm) filled with potting mixture.

At three-leaf stage, 300 mM NaCl solution was added to the pots to start the treatment for 24 h and 48 h. The same volume of water was added to the pots of the controls. The roots and shoots from three plants for each treatment and control were collected and mixed for further RNA extraction. The experiments were performed three times independently. Total RNA was extracted using the RNeasy Plant Mini Kit (Takara, Japan) according to the manufacturer’s instructions. The cDNA was synthesized with the iScript Reverse Transcription Supermix (Bio-Rad) according to the manufacturer’s instructions. cDNA was synthesized with the iScript Reverse Transcription Supermix (Bio-Rad) according to the manufacturer’s instructions. Normalization of the investigated gene transcript was relative to the reference gene GAPDH and α-tubulin. Quantitative real-time polymerase chain reaction (qPCR) was performed on a CFX96 Touch Real-Time PCR Thermal Cycler using SYBR green PCR reagent (Bio-Rad) with three technical replications. The relative expression levels of the target genes were calculated according to the comparative CT method.

### Gene domain and phylogenetic analysis

The amino acid sequence of *HvNCX* was obtained from EnsemblPlants (https://plants.ensembl.org/Hordeum_vulgare/Info/Index). Transmembrane region was predicted with Protter (http://wlab.ethz.ch/protter/start/). The gene structure was pictured with GSDS2.0 (http://gsds.gao-lab.org/). The functional domain was predicted with Smart2.0 (http://smart.embl-heidelberg.de/). The homologs sequences of *HvNCX* from other 13 species were obtained from Phytozome (https://phytozome-next.jgi.doe.gov/). The alignment of *HvNCX* amino acid sequence was performed using the software SnapGene (https://www.snapgene.com). The phylogenetic tree was constructed with the software MEGA 7 (http://www.megasoftware.net/), using a minimum-evolution method (Poisson model) with 1,000 bootstrap replicates.

### Statistical analysis

The frequency distribution analysis and correlation analysis were conducted using a statistical package IBM SPSS Statistics 20 (IBM, New York, NY, USA).

## Results

### Performance of the parents and DH lines under salinity stress

**Fig. 1:**
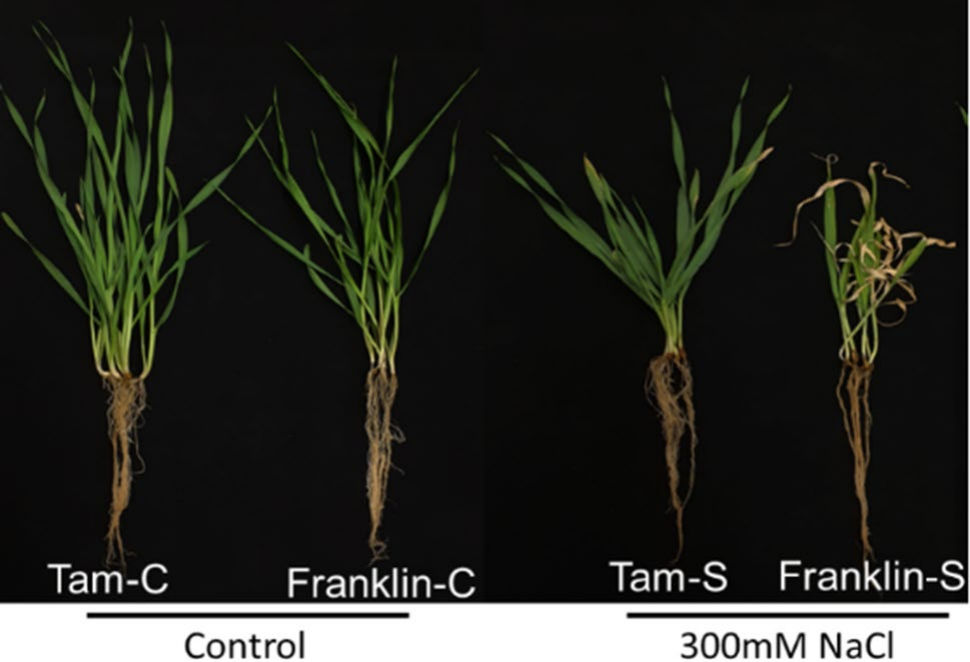
Comparison of two parental varieties Franklin and Tam407227 under control and salinity stress

Under salinity stress, two parents showed a significantly different performance, with the wild barley TAM407227 showing much better tolerance than the cultivated barley Franklin (Fig. 1). The frequency distribution of salinity damage scores and Na^+^ content under salinity stress is shown in Fig. S3. Continuous distributions were found for both traits ranging from 0.5 to 5.8 for average damage scores and 17.0–70.9 mg g^−1^ for Na^+^ contents. Franklin showed significantly higher damage scores than TAM407227, while the Na^+^ content of Franklin was only slightly higher than TAM407227. Damage scores under salinity conditions showed a significant but weak positive correlation with Na^+^ contents in leaves (*R*^2^ = 0.146) (Fig. S4).

### QTL for salinity damage score

**Fig. 2:**
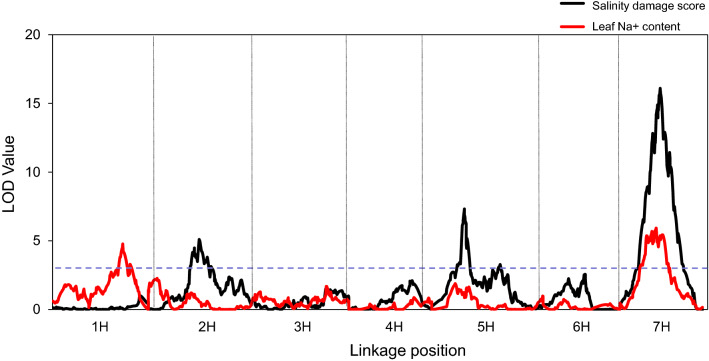
QTL mapping of locus for salinity damage score and leaf Na + content. Black line: QTL for salinity damage score under salinity stress; Red line: QTL for Na + contents under salinity stress (color figure online)

Four significant QTL were identified for salinity damage score. One (*qS2.1*) was located on chromosome 2H, determining 7.5% of the phenotypic variation. Two (*qS5.1, qS5.2*) were located on 5H, determining 5.3–11.7% of the phenotypic variation. The most significant QTL was found on 7H, explaining 27.8% of the phenotypic variation. The wild barley contributed to all tolerance alleles (Table 1, Fig. 2).

**Table 1:** QTL for salinity tolerance (S), salinity and leaf Na^+^ content under salinity stress in the DH population of Franklin × TAM407227 (only QTL with LOD value > 3.0 were shown)

QTL	Chr	Genetic position (cM)	Physical position (bp)	Flanking marker	LOD	R^2^(%)	Additive
*qS2.1*	2H	60.44–79.38	519,285,632–571,107,689	snp631-snp685	5.1	7.5	− 0.32
*qS5.1*	5H	44.54–48.92	27,420,904–438,657,462	snp1761-snp1875	7.03	11.7	− 0.40
*qS5.2*	5H	85.23–100.83	502,097,703–523,704,782	snp2009-snp2068	3.03	5.3	− 0.27
*qS7.1*	7H	52.85–61.69	73,657,568–533,861,242	snp2743-snp2853	16.1	27.8	− 0.59
*qNa1.1*	1H	94.8–106.42	479,947,385–489,727,297	snp302-snp325	4.77	11.4	33.64
*qNa7.1*	7H	43.56–61.69	55,430,357–533,861,242	snp2723-snp2853	5.92	14.1	− 37.40

### QTL for Na^+^ content in leaves

Two significant QTL were identified for Na^+^ content on 1H and 7H, respectively. The one on 1H determined 11.4% of the phenotypic variation (Table 1, Fig. 2). The other one, *qNa7.1,* was located close to *qS7.1.* and accounted for 14.1% of phenotypic variation (Table 1, Fig. 2). The allele for increasing Na^+^ contents on 7H was from Franklin, while the allele for increasing Na^+^ contents on 1H was derived from the wild barley.

### Relationship between *qNa1.1* and *HvNax4*

*qNa1.1* (physical position: 479,947,385–489,727,297 bp) was mapped to a similar position with *HvNax4* which has been reported to control Na^+^ exclusion. In a previous study, *HvNax4* was fine-mapped to a 200 Kb interval on chromosome 1H, and *HvCBL4* (physical position: 1H:488,627,624 bp), a SOS3 homolog gene, was selected as a candidate gene*.* The cDNA sequence of the *HvCBL4* allele revealed a difference in an Ala111/Thr111 amino acid substitution in the encoded protein, which may have a potential impact on the overall structure and function of barley protein (Rivandi et al. 2011). Comparison of Franklin and TAM407227 *HvCBL4* sequences revealed the same amino acid substitution difference in encoded protein (Ala111/Thr111) (Fig. S5A). According to the SNP difference, a gene CAPS marker TF-HvCBL4 was designed (Fig. S5A, B, Table S4). The PCR amplified results of the markers in 163 DH lines were the same as the nearest marker (snp314, physical position: 488,039,458 bp) of *qNa1.1*, with the largest explanation for phenotypic variation. Therefore, it is plausible to conclude that *qNa1.1* and *HvNax4* are the same QTL.

### Fine mapping of *qS7.1*

**Fig. 3:**
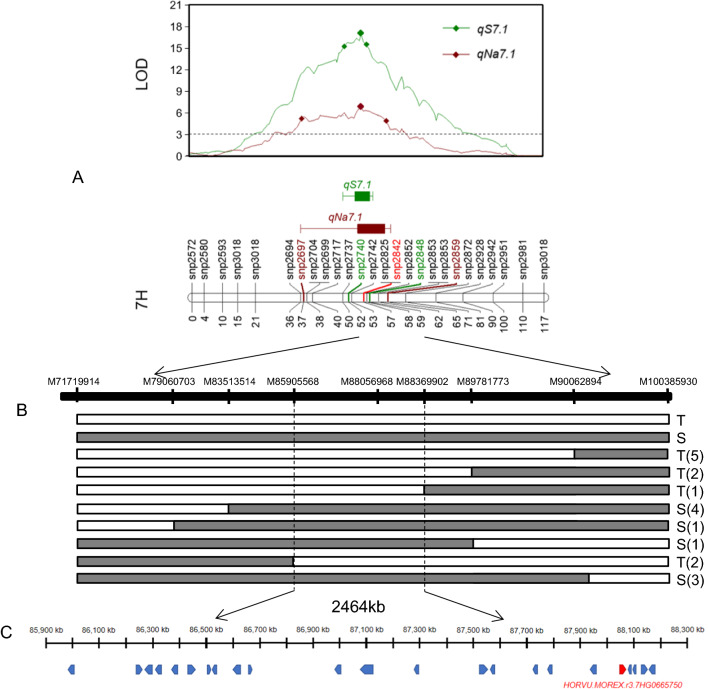
Fine mapping of *qS7.1.* A: Mapping of *qS7.1* and *qNa7.1*, the green line represents QTL for salinity damage score, the red line represents QTL for Na^+^ content; B: Marker names are indicated at the top of the column and the genotypes of the selected recombinants and the phenotype of lines of the F_3:4_ progenies derived from F_3_ plants are presented (salinity tolerance: T; salinity sensitivity: S). White rectangles indicate the homozygotes with the resistance allele of Tam, and gray rectangles indicate the homozygotes with the susceptibility allele of Franklin. The *q*S7.1 was delimited to a 2464-kb region between the M85905568 and M88369902 markers. C:High-confidence annotated genes in this region. The promising candidate gene *HORVU.MOREX.r3.7HG0665750.1* was marked using red color and others were marker with blue color (color figure online)

M7179914 and M100385930, two flanking markers for the *qS7.1*, were used to select heterozygous F_2_ plants for further self-crossing. From 1260 F_3_ lines, 19 recombinant lines were identified using the M7179914 and M100385930. The F_4_ lines derived from the 19 recombinant F_3_ lines were further genotyped with seven additional markers. By comparing salinity damage scores of different homozygous recombinants, the physical interval of *qS7.1* was narrowed to 2.46 Mb, containing 23 high-confidence annotated genes (Fig. 3).

### Candidate gene analysis of *qS7.1*

**Fig. 4:**
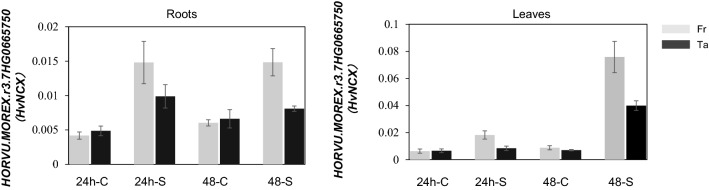
Transcription analysis of *HvNCX* response to salt stress in root and leaves after 24 h and 48 h salinity treatment. 24 h-C: 24 h under control conditions; 24 h-S: 24 h under salinity stress conditions; 48 h-C: 48 h under control conditions; 48 h-S: 48 h under salinity stress conditions

**Fig. 5:**
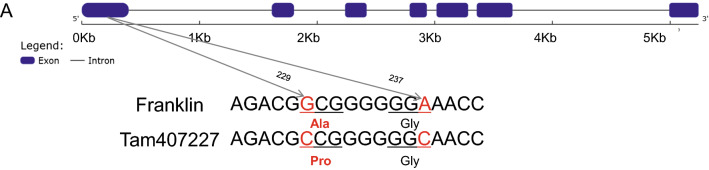
Sequencing analysis of *HvNCX*

qPCR was performed in the two parents for the 23 high-confidence annotated genes using roots and leaves sampled 24 h and 48 h after salinity treatment (Table S5). Among these genes, the expression levels of five genes increased or decreased significantly in response to salt stress in roots or leaves of both parents (Fig. 4, S6). The five genes are *HORVU.MOREX.r3.7HG0665490*, *HORVU.MOREX.r3.7HG0665710*, *HORVU.MOREX.r3.7HG06657730*, *HORVU.MOREX.r3.7HG0665750* and *HORVU.MOREX.r3.7HG0665830*, encoding myosin-1, mitochondrial transcription termination factor-like, heavy metal transport/detoxification superfamily protein, sodium/calcium exchanger family protein and mitochondrial transcription termination factor-like, respectively. Among them, only two genes, (*HORVU.MOREX.r3.7HG0665750* and *HORVU.MOREX.r3.7HG0665490*), were highly induced by salinity stress and showed significant differences between the two parents, with the expression level of *HORVU.MOREX.r3.7HG0665750* (*HvNCX*) in the roots of the sensitive parent Franklin being more than tenfold higher than the control after 48 h of salt treatment (Fig. 4). The sequence analysis of *HORVU.MOREX.r3.7HG0665490* showed no difference (Fig. S7) while *HvNCX* revealed two single nucleotide substitutions in the first exon region and only one result in Ala and Pro amino acid substitution in encoded proteins, which is located on the transmembrane domain (Fig. 5, S8A). The sequences alignment of *HvNCX* in 20 cultivars (representatives of global barley diversity) using pan-genome showed that the Ala/Pro substitution is unique in Tam40722 (Fig. S9). Furthermore, the promoter sequencing analysis of *HvNCX* showed multiple nucleotide substitutions and deletions/insertions between the two parents (Fig. S10). The expression and sequencing results indicated that *HvNCX* is most likely the candidate gene for *qS7.1.*

### Domain and phylogenetic analysis of *HvNCX*

The genomic sequence of *HvNCX* contains 5,241 bp with six introns and seven exons, and the full length of its complementary DNA (cDNA) is 1,734 bp, encoding a polypeptide of 578 amino acids (Fig. 5). The Protter software was used to predict the protein structure of *HvNCX*, and the results showed that *HvNCX* contained 10 transmembrane domains (Fig.S8A). The Smart software revealed that *HvNCX* contained two sodium/calcium exchanger domains and two calcium-binding motifs (Fig. S8B). Phylogenetic analysis showed that *HvNCX* had 54.9% to 95.7% amino acid identity to 25 members of *NCX* subfamily transporters from 13 plant species, with the highest sequence similarity to *NCX* from *thinopyrum intermedium* (Fig. S11). *Thinopyrum intermedium* is one of the wild relatives of wheat with many excellent abiotic stress tolerance genes.

## Discussion

In barley, only two QTL for Na^+^ exclusion, *HvNax4* and *HvNax3*, have been fine-mapped so far, and the genes *HVP10* and *HvCBL4* are regarded as the candidate genes, respectively (Rivandi et al. 2011; Shavrukov et al. 2013)*.* The gene *HvCBL4 (*physical position: 488,627,624 bp*)* was located within the region of *qNa1.1* (physical position: 479,947,385–489,727,297 bp), and the sequencing analysis of *HvCBL4* in parents revealed that the amino acid substitution difference is the same as the previous study on *HvNax4* (Rivandi et al. 2011), suggesting *qNa1.1* and *HvNax4* were more likely the same QTL. The *qS7.1* (physical position: 73,657,568–533,861,242) and *qNa7.1* (physical position:55,430,357–533,861,242) were mapped to a similar position of the reported gene *HVP10 (*physical position: 54,116,042 bp) (Shavrukov et al. 2013). As a candidate gene for *HvNax3,* the function of *HVP10* has been confirmed by Fu et al. 2022). *HVP10* is mainly expressed in roots and was upregulated under salt stress, and *HVP10* knockdown (RNA interference) and knockout (CRISPR/Cas9 gene editing) barley plants showed greatly inhibited growth and higher shoot Na^+^ concentration, Na^+^ transportation rate and xylem Na^+^ loading than the wild-type plants (Fu et al. 2022). To confirm the relationship between *qS7.1 and HVP10,* the sequences and the transcriptive levels of *HVP10* in two parents were compared. The expression level of *HVP10* was not significantly changed in response to salt treatment, and the CDS sequencing showed no significant differences between the two parents (Fig. S12). Further fine mapping also supported that *qS7.1* was closely linked with *HvHVP10.*

Na^+^ exclusion is one of the major mechanisms for plants tolerance to salinity (Fan et al. 2015; Siahsar and Narouei 2010). The maintenance of ion homeostasis is considered to be critical in determining salinity tolerance. Cytosolic ion homeostasis under salt stress implies the complex and orchestrated operation of numerous transport systems involved in ion uptake, sequestration, and long-distance transport (Almeida et al. 2017). In cereal crops, many QTL or gene for salinity tolerance and Na^+^ exclusion have been identified. These include a HAK family ion transporter *ZmHAK4* which confers natural variation of salt tolerance in maize (Zhang et al. 2019); *TaRN1* and *TaRN2,* two novel candidate genes for salinity tolerance in wheat showing different expression patterns in contrasting salt-tolerant wheat genotypes (Li et al. 2021); and *GmSALT3* which encodes a protein from the cation/H^+^ exchanger family in soybean (Qu et al. 2021). In this study, based on the fine mapping, sequencing and expression analysis, Na^+^/Ca^2+^ exchanger was identified as a promising candidate gene for improving salinity stress tolerance located at *qS7.1* loci.

The NCX is an ion transporter that exchanges Na^+^ and Ca^2+^ in either Ca^2+^ efflux or Ca^2+^ influx mode, depending on membrane potential and transmembrane ion gradients (Iwamoto 2006). The functions of the NCX family are well understood in humans, but its functional role remains to be discovered in plants. Though NCX has a low affinity toward Ca^2+^, it can transport Ca^2+^ at a very high speed, up to 5000 calcium ions per second in a short period of time (Carafoli et al. 2001). In mammalian systems, the NCX proteins mediate an electrogenic exchange of the three Na^+^ for one Ca^2+^, while the net ion flux can occur in either forward (Ca^2+^ exclusion-Na^+^ entry coupling) or reverse (Na^+^ exclusion -Ca^2+^ entry) (Khananshvili 2014). High extracellular Na^+^ levels trigger a substantial Na^+^ influx and Ca^2+^ loss and Na^+^/Ca^2+^ exchanger 1 (*NCX1*) is able to sense Na^+^ and plays a critical role in high salt-triggered Na^+^ influx, concomitant Ca^2+^ efflux in macrophages (Neubert et al. 2020). Twenty-two NCX proteins encoded by fifteen genes in rice and sixteen NCX proteins encoded by thirteen genes in Arabidopsis have been identified. Among them, *OsNCX3*, *OsNCX10* and *OsNCX15* exhibited predominantly upregulation in response to salinity and *AtNCX7*, *AtNCX9*, *AtNCX10*, *AtNCX12* and *AtNCX13* were highly induced by salt stress in both root and shoot (Singh et al. 2015). Arabidopsis NCX-like (*AtNCL*), encoding a protein with an NCX-like structure, has the ability to bind Ca^2+^ and is involved in salt stress in Arabidopsis by regulating Ca^2+^homeostasis. Compared to wild type, calcium content in whole *atncl* mutant seedlings was higher and the level of free Ca^2+^ in the cytosol and Ca^2+^ flux at the root tips of *atncl* mutant plants required a longer recovery time following NaCl stress. Loss-of-function *atncl* mutants show higher salinity tolerance than wild-type or *AtNCL* transgenic over-expression lines (Wang et al. 2012), indicating that this gene negatively regulates salt tolerance in *Arabidopsis*. This is consistent with our qPCR results. We observed a strong expression of this gene in sensitive variety (Fig. 5), demonstrating a negative regulatory relationship between NCX and plant salinity tolerance. A tonoplast-localized *AtNCL* shows the ability to deplete cytosolic Na^+^ into the vacuole in exchange of Ca^2+^ efflux and suppress yeast vacuolar Na^+^/H^+^ transporter NHX mutants (Li et al. 2016). *AtNCL* mutants (*atncl-1*, *atncl-2*) display reduced Ca^2+^ accumulation under Ca^2+^ stress conditions and accumulate more Na^+^ under NaCl stress, thus being more sensitive to salt stress (Li et al. 2016). NCX works in both directions depending upon the gradient generated by Ca^2+^ and Na^+^ concentrations inside the cell (Yu et al. 1997; Wolf et al. 2001). When specific salt stress conditions are encountered, *AtNCL* appears to function as a Na^+^ transporter (Li et al. 2016). These two studies provided convincing evidence for functions of *AtNCL* in transporting Na^+^/Ca^2+^ under salt stress. *NCX* transporters have received much less attention although they may play an important role in salinity tolerance by regulating ion homeostasis under salinity conditions. In this study, Ala amino acid at position 77 is substituted by Pro in the wild barley TAM407227, which is unique from all other cultivated barley genotypes (Fig. 5, Fig. S9). Further studies are needed to investigate how the Ala and Pro amino acid substitution influences the gene function of *HvNCX* using over-expression and genome editing technology.

Plant breeders have made some progress in producing salt-tolerant lines through modern molecular biology methods. Rice salinity tolerance genes *SKC1* and *HST* have been successfully used in salt tolerance breeding. An HKT-type transporter, *SKC1*, was cloned by map-based cloning strategies, which is preferentially expressed in the parenchyma cells surrounding the xylem vessels and involved in regulating K^+^/Na^+^ homeostasis under salt stress (Ren et al. 2005). Molecular marker-assisted selection for *SKC1* has been carried out through marker-assisted backcross, and *SKC1* can significantly reduce yield losses under salt stress (3–26%) (Bimpong et al. 2016). *HST* is a salt-tolerant gene identified from a salt-tolerant mutant in rice, encoding an MYB-type transcription. *HST* was introduced into a salt-sensitive variety by molecular breeding in just two years, and the grain yield per *hst1* plant in salt-treated plots was more than double that of the WT plants (Takagi et al. 2015). Field trials on saline soils showed that *TmHKT1;5-A* significantly reduces Na^+^ accumulation in leaves and increases durum wheat grain yield by 25% (Munns et al. 2012). Currently, the use of salt-tolerant wild relatives to improve crop salt tolerance has become a hot topic (Razzaq et al. 2021). Wild barley is adapted to a wide range of extreme latitudes, altitudes, climates (warm and cold), and soils. Comparison of the genomes of cultivated genotype Morex and wild barley showed that wild barley contained more genes of biotic and abiotic stress resistance and tolerance (Liu et al. 2020). Several important genes or QTL have been identified in wild barley. *Rph15* is a gene derived from wild barley conferring resistance to leaf rust (Chen et al. 2021). A novel QTL *qRYM-2Ha* contributing to barley yellow mosaic resistance was identified in wild barley (Pan et al. 2021). Association and expression analysis revealed that Tibetan wild barley offers elite alleles of *HvHKT1* and *HvHKT2* conferring salinity tolerance (Qiu et al. 2011). However, up to now, no gene has been successfully used in barley salt-tolerant breeding. In this study, two major QTL for salinity tolerance on chromosomes 5H and 7H were identified with the wild barley TAM407227 contributing both tolerance alleles. For the QTL associated with Na^+^ exclusion, Franklin contributes the tolerance allele on 1H, and TAM407227 contributes the tolerance allele on 7H. Of the two QTL for Na^+^ content, the one on 7H showed a significant correlation to salinity tolerance and thus can be an important source for use in a breeding program. The candidate gene *HvNCX* may represent a new type of Na^+^/Ca^2+^ transporter in higher plants and opens new prospects for improving salinity tolerance.

## Supplementary Information

Below is the link to the electronic supplementary material.

Supplementary file 1 (PPTX 1183 KB)

Supplementary file 2 (PDF 748 KB)

Supplementary file 3 (XLS 7102 KB)

Supplementary file 4 (DOCX 25 KB)

## Data Availability

The datasets supporting the conclusions of this article are included within the article and its additional files. Sub-section of all DH lines can be obtained from the corresponding author, Prof Meixue Zhou, TIA, University of Tasmania, under Material Transfer Agreement.
