# Supplementary material for: HvNCX, a prime candidate gene for the novel qualitative locus qS7.1 associated with salinity tolerance in barley
Source: Theor Appl Genet. 2023 Jan 19;136(1):9. doi: 10.1007/s00122-023-04267-4 (PMC9852152; doi:10.1007/s00122-023-04267-4)
Supplement: Supplementary file 1 — (PPTX 1183 KB) [file 122_2023_4267_MOESM1_ESM.pptx]

## Slide 1
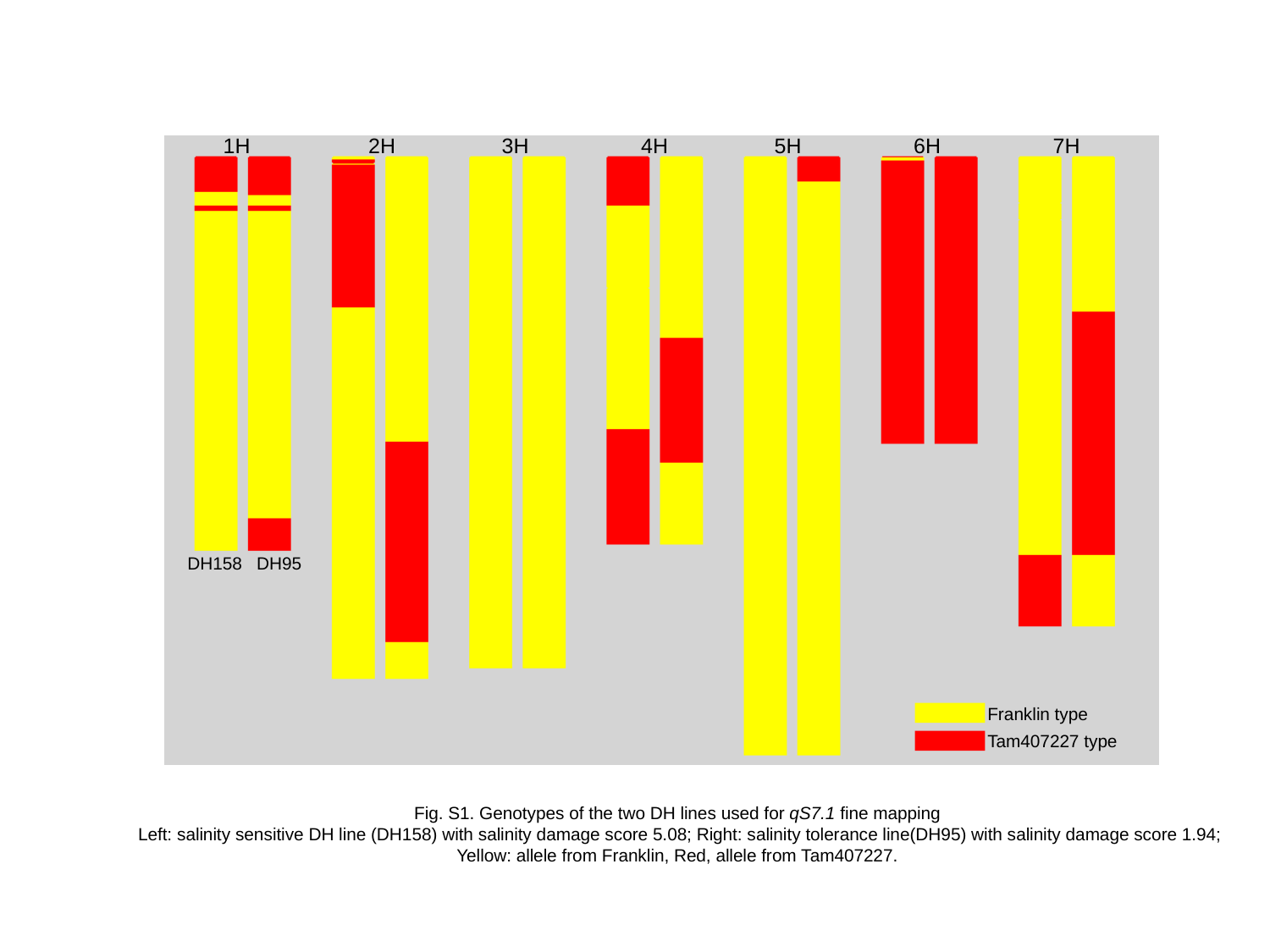

1H 2H 3H 4H 5H 6H 7H
DH158 DH95
Franklin type
Tam407227 type
Fig. S1. Genotypes of the two DH lines used for qS7.1 fine mapping
 Left: salinity sensitive DH line (DH158) with salinity damage score 5.08; Right: salinity tolerance line(DH95) with salinity damage score 1.94;
Yellow: allele from Franklin, Red, allele from Tam407227.

## Slide 2
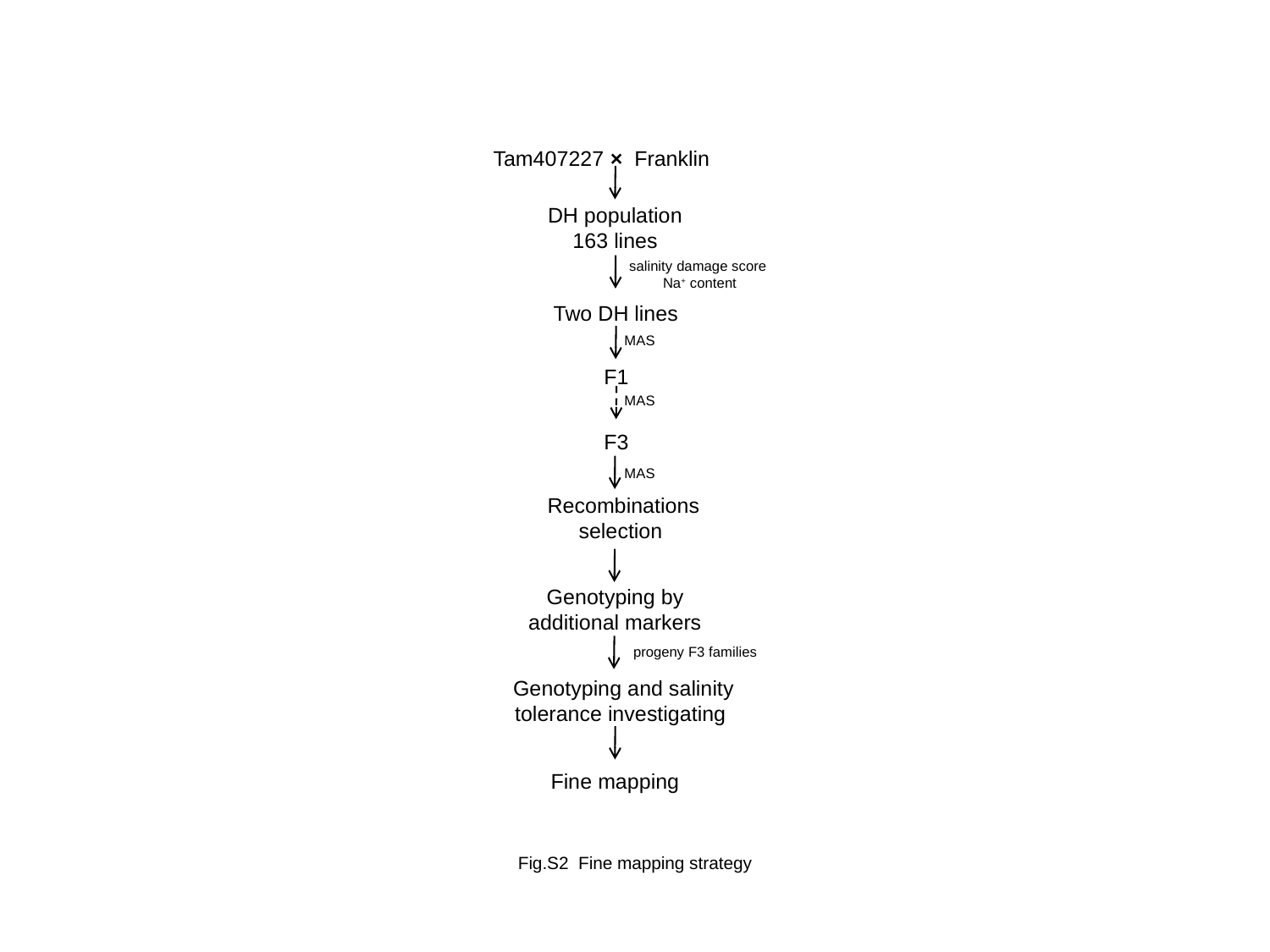

# Tam407227 × Franklin
DH population
163 lines
salinity damage score
 Na+ content
Two DH lines
MAS
F1
MAS
F3
MAS
Recombinations selection
Genotyping by additional markers
progeny F3 families
Genotyping and salinity tolerance investigating
Fine mapping
Fig.S2 Fine mapping strategy

## Slide 3
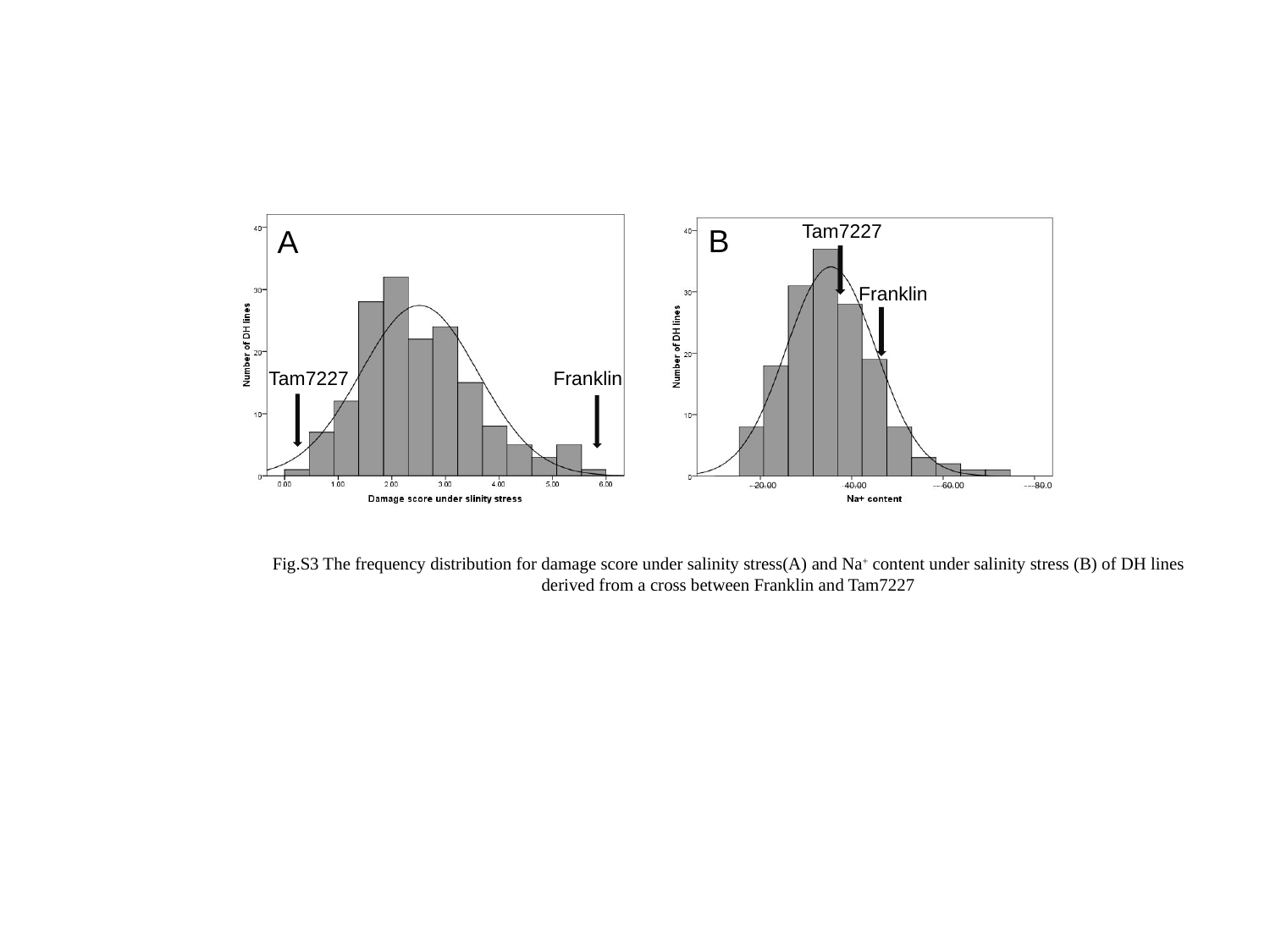

Franklin
Tam7227
Tam7227
Franklin
B
20.00 40.00 60.00 80.0
A
Fig.S3 The frequency distribution for damage score under salinity stress(A) and Na+ content under salinity stress (B) of DH lines
derived from a cross between Franklin and Tam7227

## Slide 4
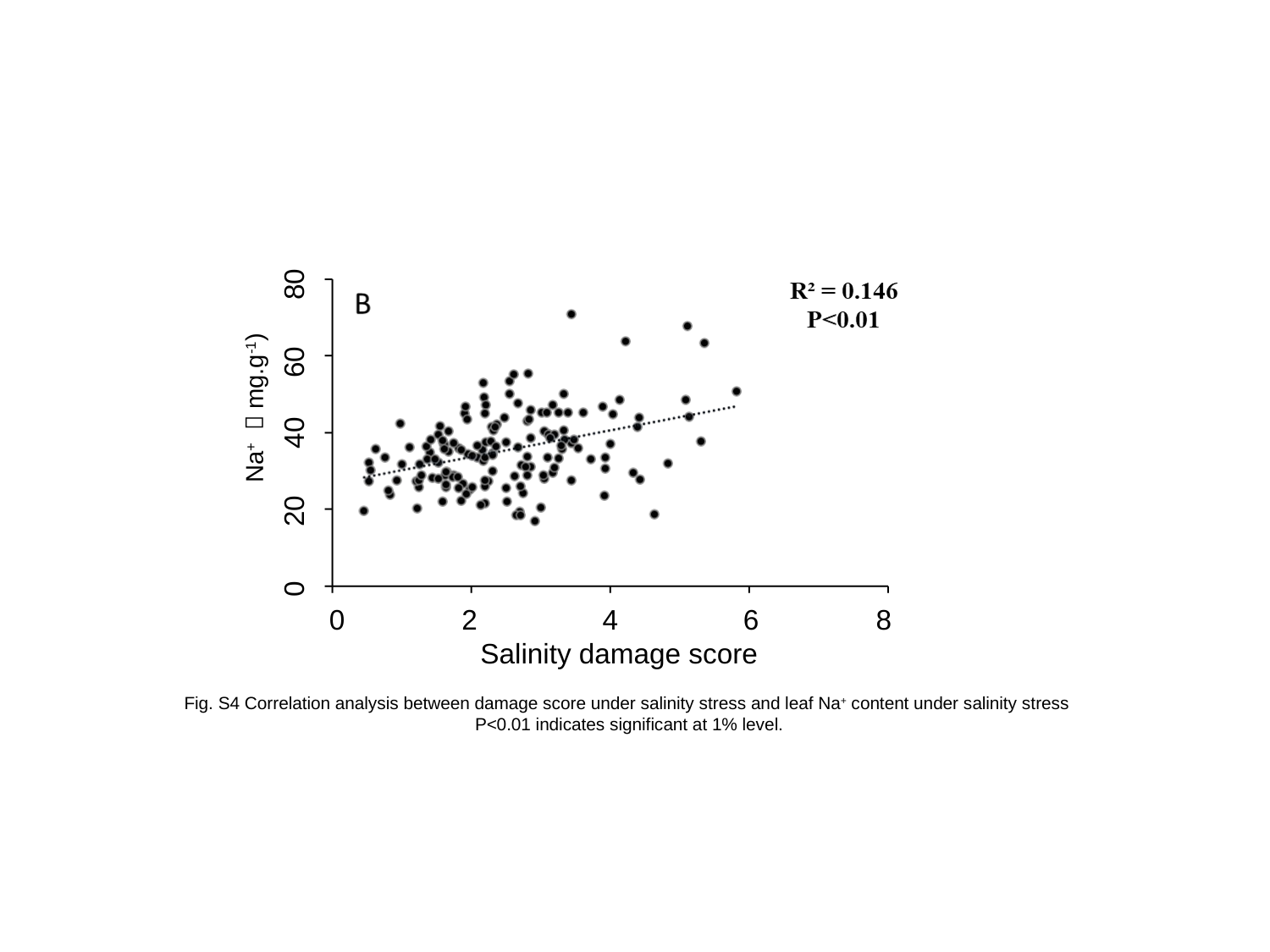

#
Na+ （mg.g-1)
0 20 40 60 80
0 2 4 6 8
Salinity damage score
Fig. S4 Correlation analysis between damage score under salinity stress and leaf Na+ content under salinity stress
 P<0.01 indicates significant at 1% level.

## Slide 5
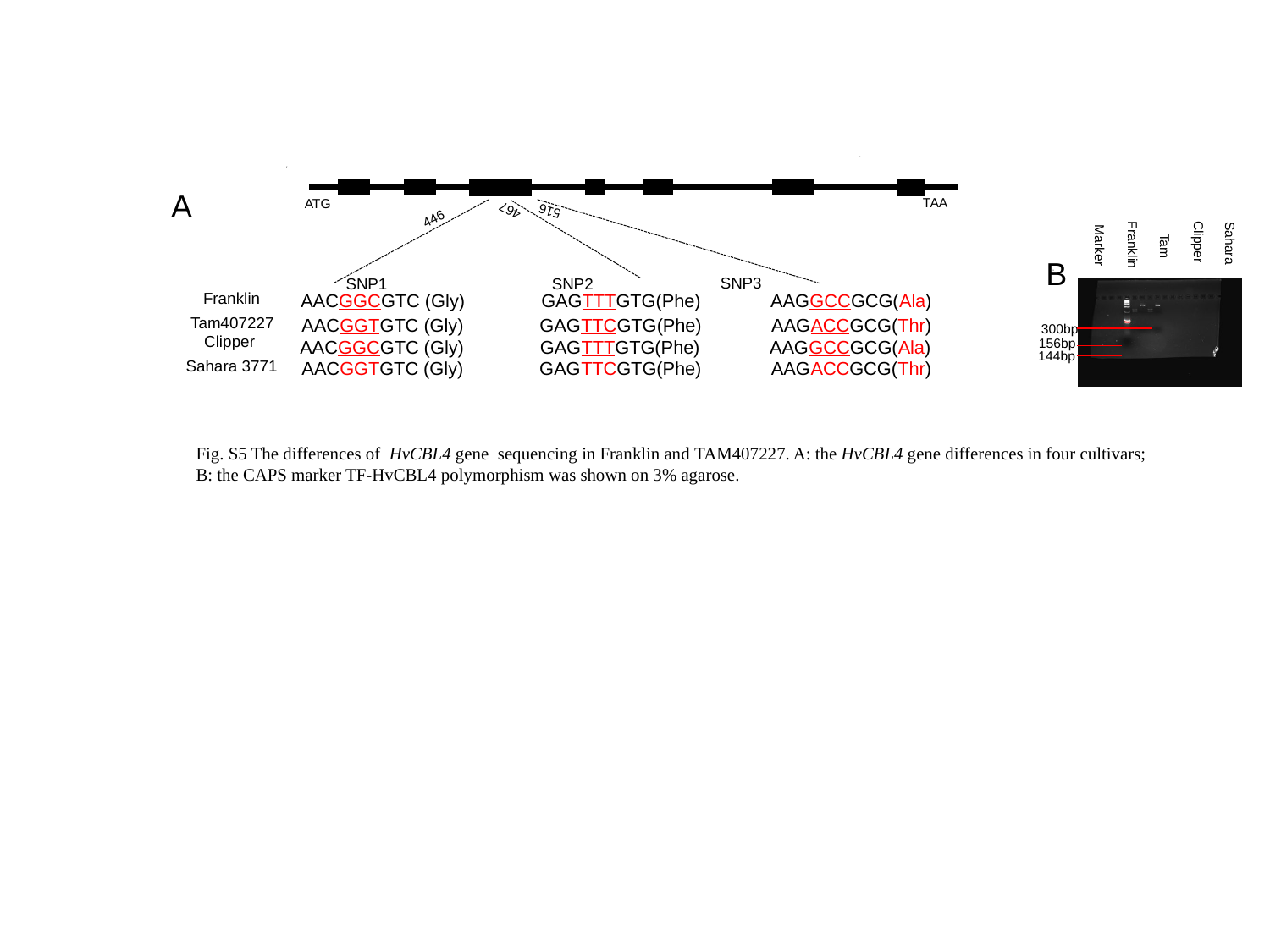

3’
5’
A
TAA
ATG
467
516
446
Clipper
Sahara
Marker
Franklin
Tam
B
B
SNP3
SNP1
SNP2
Franklin
AACGGCGTC (Gly) GAGTTTGTG(Phe) AAGGCCGCG(Ala)
Tam407227
AACGGTGTC (Gly) GAGTTCGTG(Phe) AAGACCGCG(Thr)
300bp
Clipper
156bp
AACGGCGTC (Gly) GAGTTTGTG(Phe) AAGGCCGCG(Ala)
144bp
Sahara 3771
AACGGTGTC (Gly) GAGTTCGTG(Phe) AAGACCGCG(Thr)
Fig. S5 The differences of HvCBL4 gene sequencing in Franklin and TAM407227. A: the HvCBL4 gene differences in four cultivars; B: the CAPS marker TF-HvCBL4 polymorphism was shown on 3% agarose.

## Slide 6
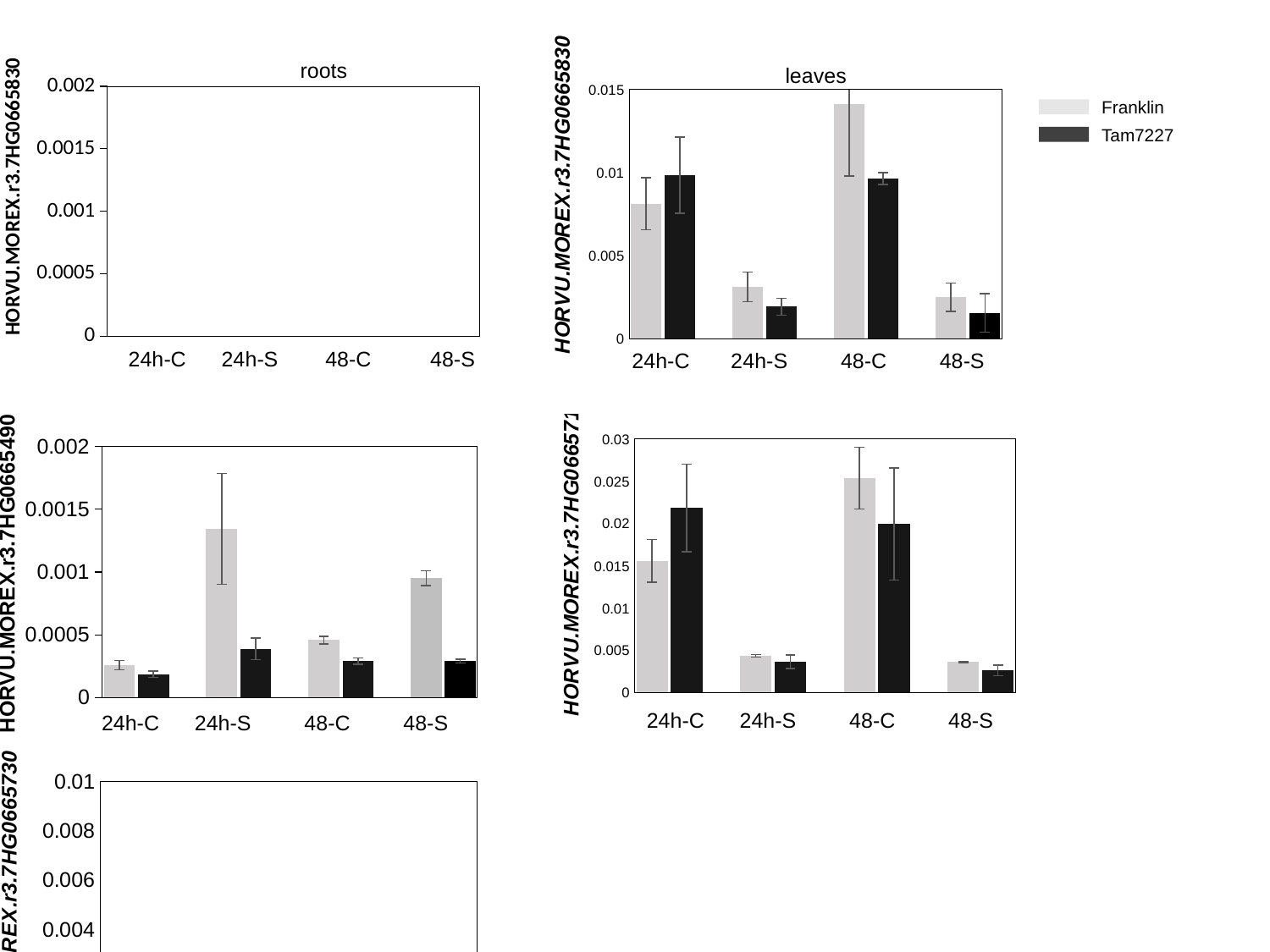

### Chart
| Category | |
|---|---|
| F-24ck | 0.00813277257074092 |
| T-24ck | 0.00985478277409289 |
| | None |
| F-24h | 0.00311573696688442 |
| T-24h | 0.00192043446447068 |
| | None |
| F-48ck | 0.0141171203016994 |
| T-48ck | 0.00964489196353012 |
| | None |
| F-48h | 0.00249328185782588 |
| T-48h | 0.00155630327469288 |
### Chart
| Category | |
|---|---| roots
24h-C 24h-S 48-C 48-S
leaves
24h-C 24h-S 48-C 48-S
Franklin
Tam7227
### Chart
| Category | |
|---|---|
### Chart
| Category | |
|---|---|
| F-24ck | 0.0155847670925866 |
| T-24ck | 0.0218718749649077 |
| | None |
| F-24h | 0.00434502732931718 |
| T-24h | 0.00366335377992862 |
| | None |
| F-48ck | 0.0253780335846796 |
| T-48ck | 0.0199487338600893 |
| | None |
| F-48h | 0.00361596860754112 |
| T-48h | 0.00260959704031629 |
### Chart
| Category | |
|---|---|
| F-24ck | 0.00603927974498164 |
| T-24ck | 0.00653449731173302 |
| | None |
| F-24h | 0.00301142972237601 |
| T-24h | 0.00379883070312349 |
| | None |
| F-48ck | 0.00690586248359746 |
| T-48ck | 0.00729762913055413 |
| | None |
| F-48h | 0.00326713687120412 |
| T-48h | 0.00287889522640884 | 24h-C 24h-S 48-C 48-S
 24h-C 24h-S 48-C 48-S
 24h-C 24h-S 48-C 48-S
Fig.S6 Transcription analysis of genes in response to salt stress in roots and leaves after 24 h and 48h salinity treatment
24h-C: 24 hours under control conditions; 24h-S: 24 hours under salinity stress conditions; 48h-C: 48 hours under control conditions; 48h-S: 48 hours under salinity stress conditions.

## Slide 7
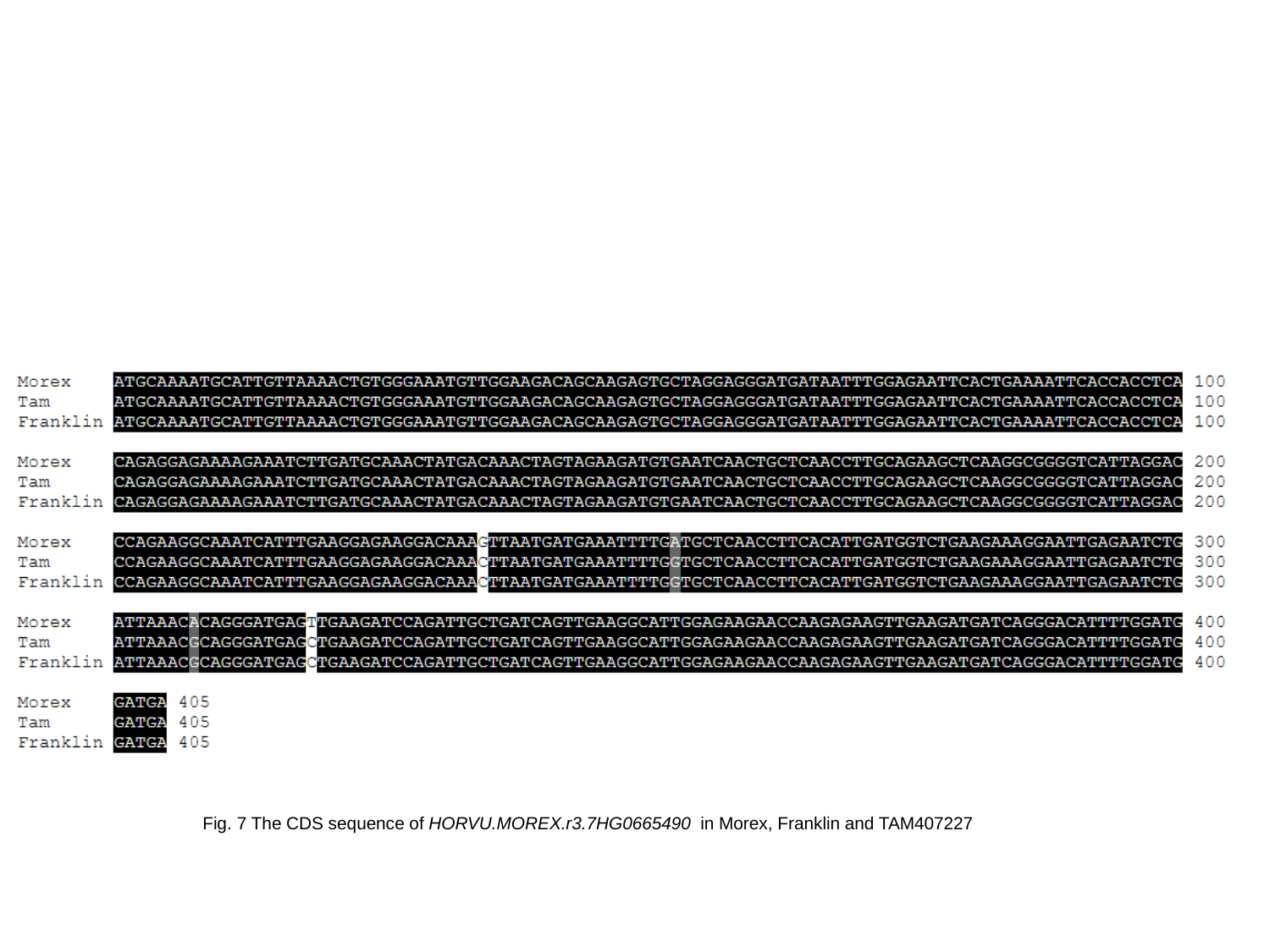

Fig. 7 The CDS sequence of HORVU.MOREX.r3.7HG0665490 in Morex, Franklin and TAM407227

## Slide 8
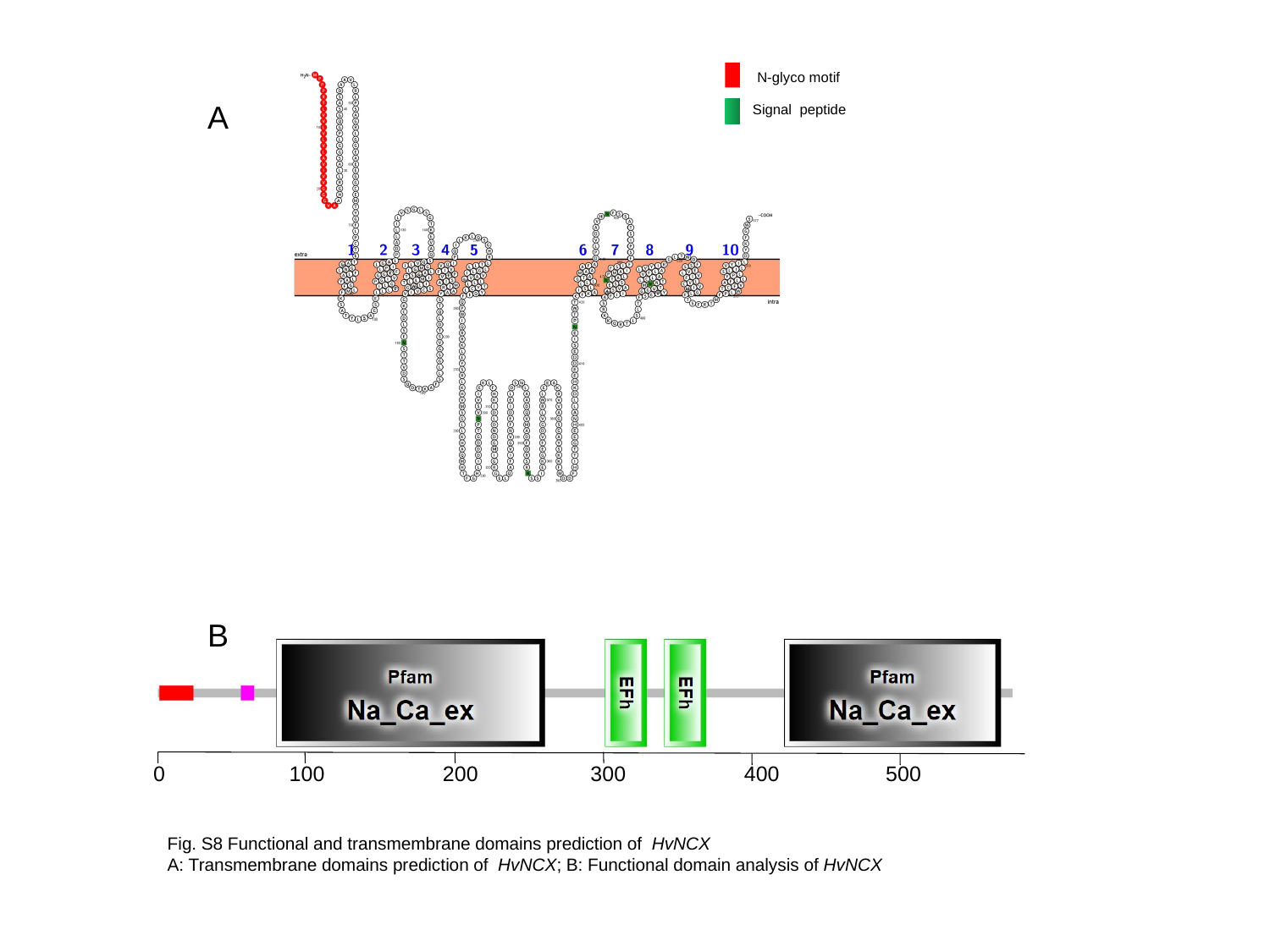

N-glyco motif
Signal peptide
A
B
 0 100 200 300 400 500
Fig. S8 Functional and transmembrane domains prediction of HvNCX
A: Transmembrane domains prediction of HvNCX; B: Functional domain analysis of HvNCX

## Slide 9
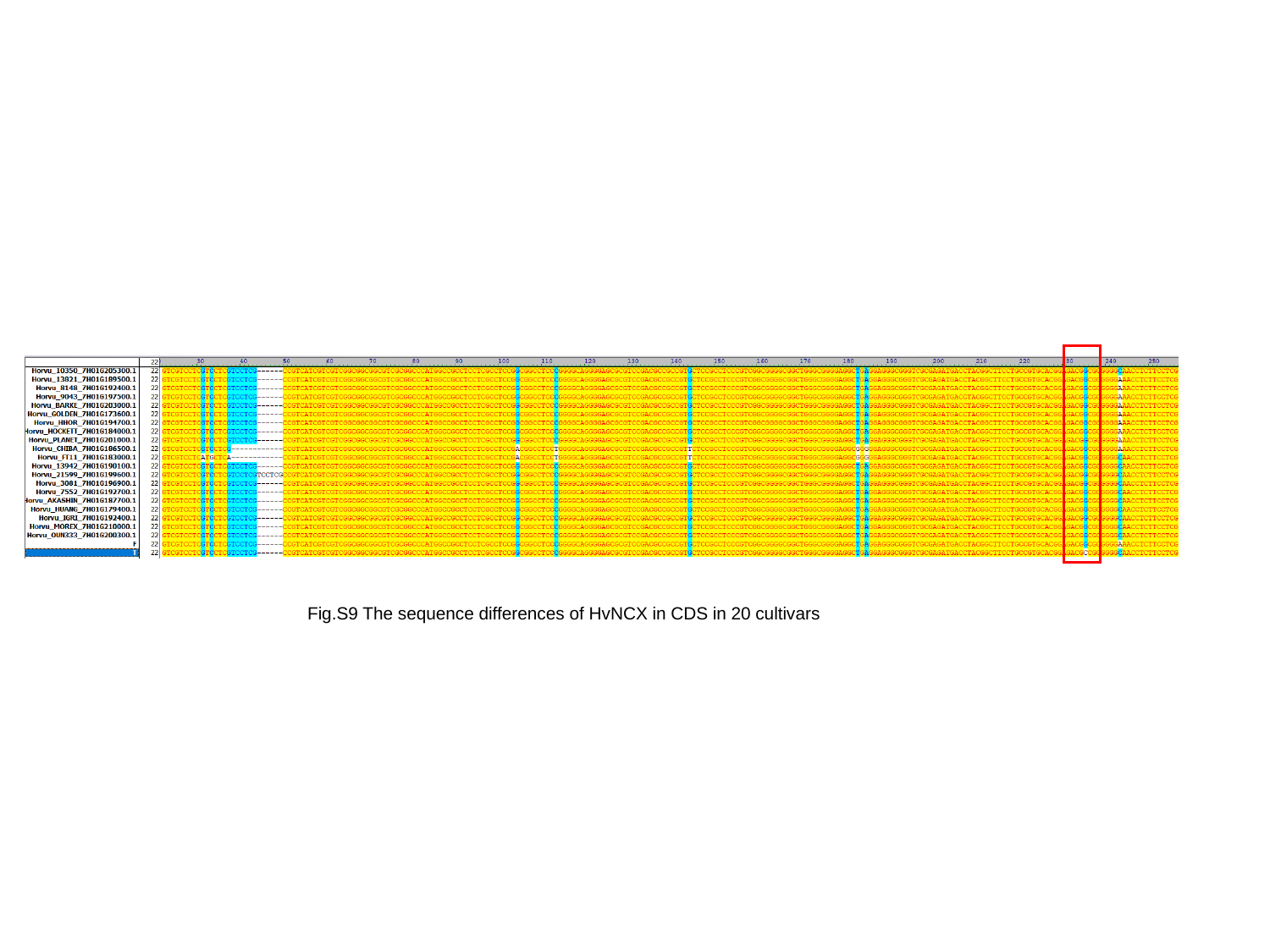

#
Fig.S9 The sequence differences of HvNCX in CDS in 20 cultivars

## Slide 10
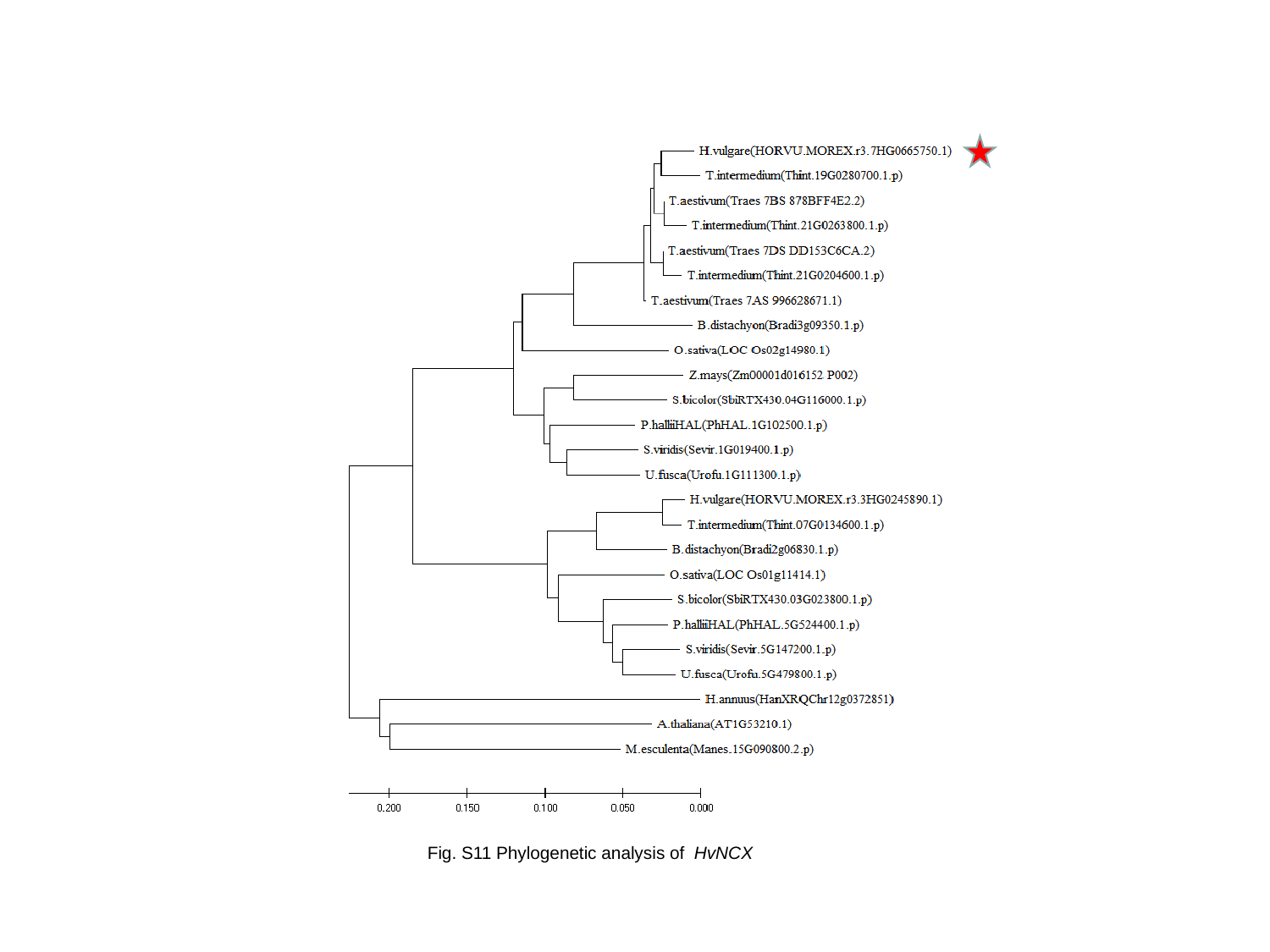

Fig. S11 Phylogenetic analysis of HvNCX

## Slide 11
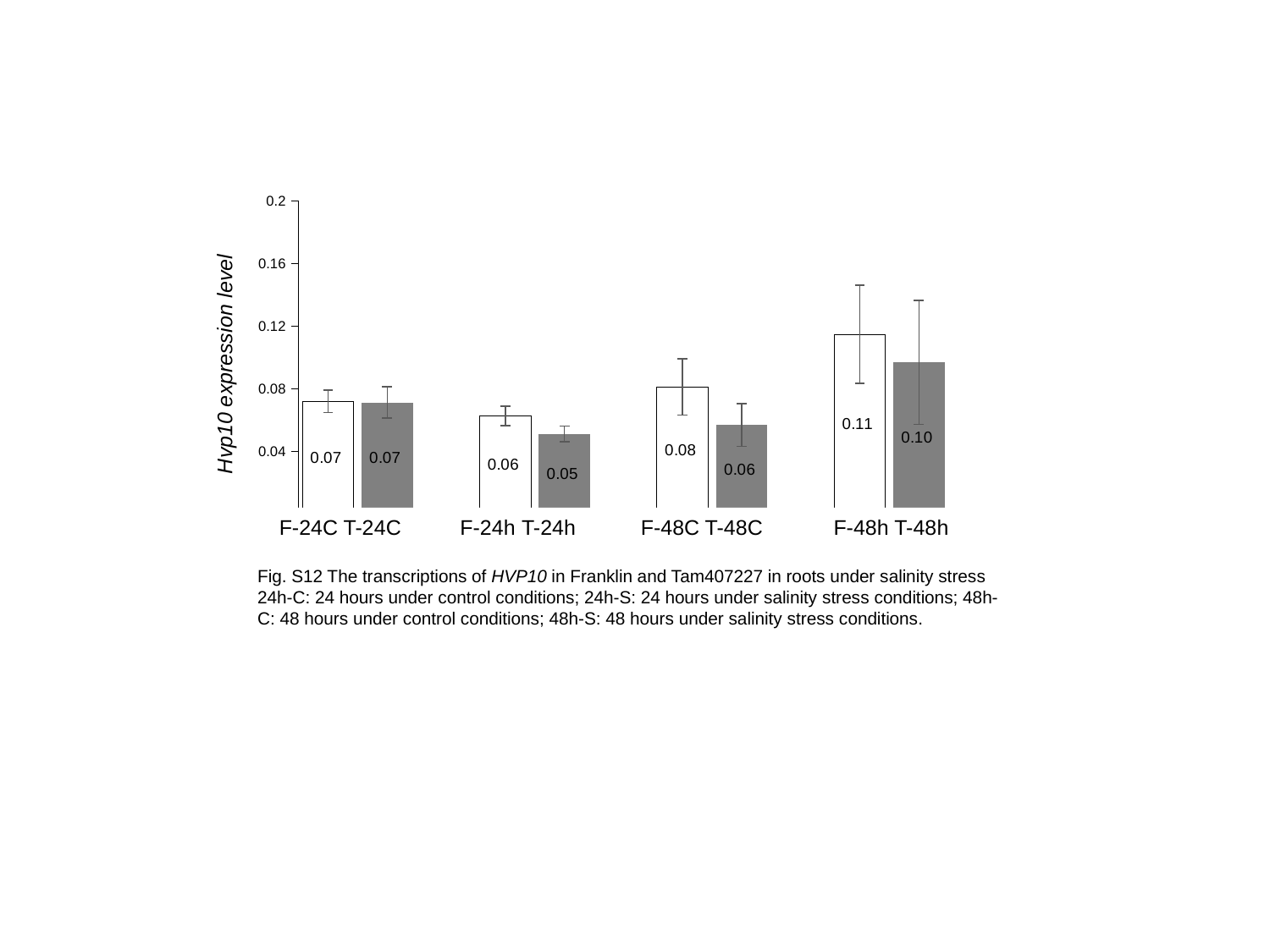

### Chart
| Category | |
|---|---|
| F-24ck | 0.07192 |
| T-24ck | 0.07125 |
| | None |
| F-24h | 0.06249 |
| T-24h | 0.05111 |
| | None |
| F-48ck | 0.0811485 |
| T-48ck | 0.0568 |
| | None |
| F-48h | 0.11485 |
| T-48h | 0.0968 |F-24C T-24C F-24h T-24h F-48C T-48C F-48h T-48h
Fig. S12 The transcriptions of HVP10 in Franklin and Tam407227 in roots under salinity stress
24h-C: 24 hours under control conditions; 24h-S: 24 hours under salinity stress conditions; 48h-C: 48 hours under control conditions; 48h-S: 48 hours under salinity stress conditions.
