# Supplementary material for: HvNCX, a prime candidate gene for the novel qualitative locus qS7.1 associated with salinity tolerance in barley
Source: Theor Appl Genet. 2023 Jan 19;136(1):9. doi: 10.1007/s00122-023-04267-4 (PMC9852152; doi:10.1007/s00122-023-04267-4)
Supplement: Supplementary file 2 — (PDF 748 KB) [file 122_2023_4267_MOESM2_ESM.pdf]

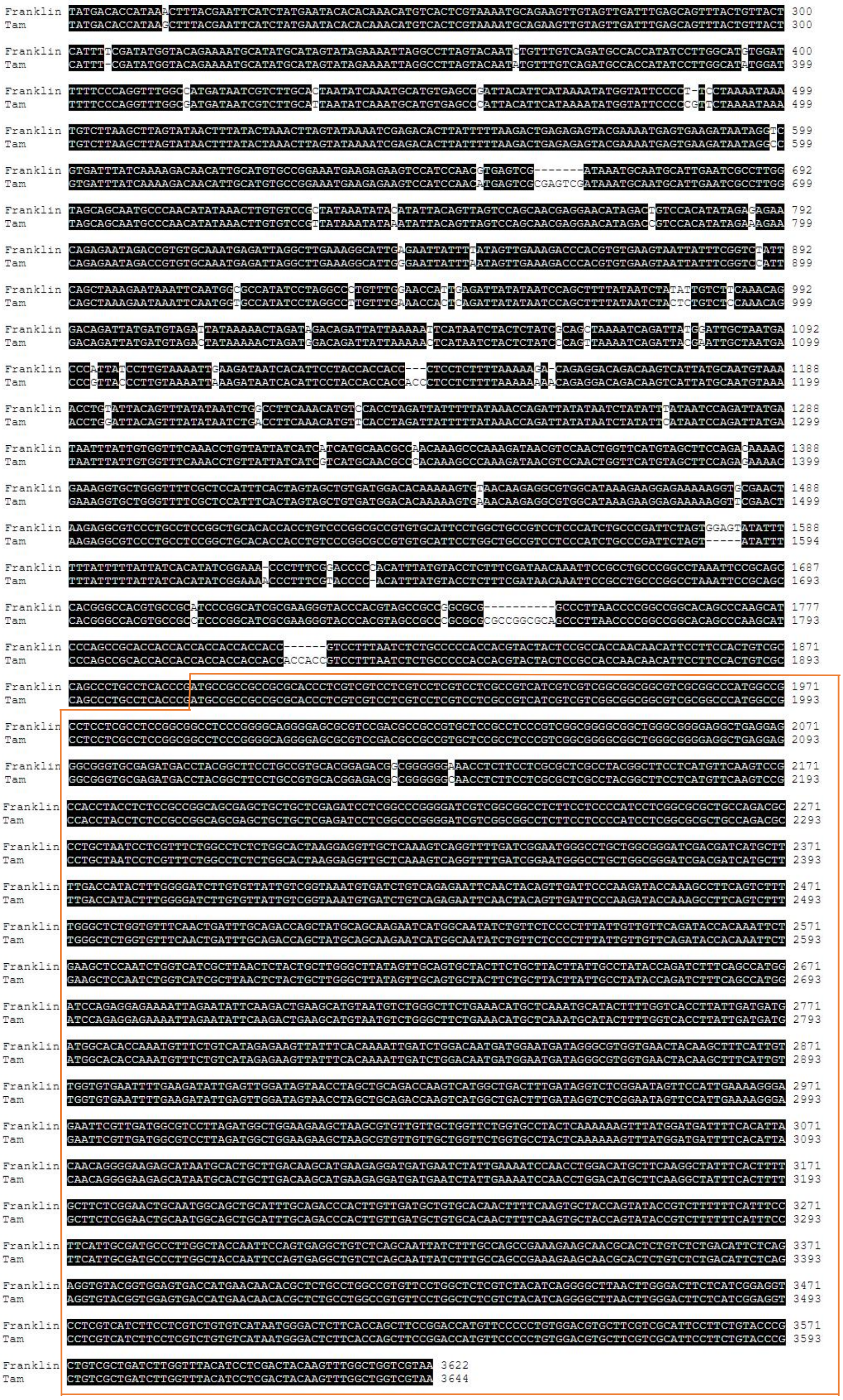

Fig. S5 The promoter and CDS sequence of *HvNCX* in Franklin and TAM407227
