# Supplementary material for: HvNCX, a prime candidate gene for the novel qualitative locus qS7.1 associated with salinity tolerance in barley
Source: Theor Appl Genet. 2023 Jan 19;136(1):9. doi: 10.1007/s00122-023-04267-4 (PMC9852152; doi:10.1007/s00122-023-04267-4)
Supplement: Supplementary file 4 — (DOCX 25 KB) [file 122_2023_4267_MOESM4_ESM.docx]

Table S4 Primers used in this study

| Primer name | Forward Sequence (5'-3') | Reverse Sequence (5'-3') |
| --- | --- | --- |
| TF-CBL4 | TGTTCAGGACCAGCAAAGGG | ATGCAACTACAAAGTGGGATG |
| TF-HVP10 | CATTGTTGGAACCCTCTTTG | AACTCAAAGGAAGAAAGAAG |
| 83513514 | GGGACTGGGTGTTGGATAC | GCGGAGGGAGGATTAACTC |
| 85905568 | TTCACATAGGAGAAAGCCCAC | CCGTGGCAAGAGGAAGAAGAC |
| 79060703 | CTCGATGGCAGAACCTAGATG | GCACCGCACAGAAGGACTTAC |
| 89781773 | GGCAGAAGTGGGAATAGAGG | GTCGAAGGCTGGTTGATACG |
| 90062894 | CATCGCTGCATTGTACTCCC | GCAAAGAAGTGAATTGCAAAG |
| 71719914 | TTCAGGACTGGGAGTTGTTGC | CGAGTGGCGTATTATTTCATCT |
| 100385930 | ATTCCTTGATCGTCGTAACC | CTGATTTCAACCATGACATTTTG |
| HvNCX-CDS | ATGCCGCCGCCGCGCACCCTCGT | TTACGACCAGCCAAACTTGTAGTC |
| HvNCX-RT | ATTCGTTGATGGCGTCCTTAG | CTCTTCCCCTGTTGTAATGTG |
| HvNCX-promoter1 | GATTAGGCTTGAAAGGCATTGGG | CAGGGCTGGCGACAGTGGAAGG |
| HvNCX-promoter2 | CAAACACATTGAACTATTGAGAC | TGTTCCTCGTTGCTGGACTAAC |
| HORVU.MOREX.r3.7HG0665490-CDS | ATCTATCAACATACTTGGGTTGAAC | AACTGTGGTTTTGTTGGTTGGGTAG |
| HORVU.MOREX.r3.7HG0665410-RT | TCGCCACTCACCACCACATG | GCAAGTAGAGCGGCAGGATC |
| HORVU.MOREX.r3.7HG0665460-RT | GCGAGGGAGTATTATTGCTTCA | ACCTTTTGATTCACTGGCTTTG |
| HORVU.MOREX.r3.7HG0665480-RT | GGATTAGATTCAAGTGGCTGTG | CTTCTCAACACTGAACCGCAAC |
| HORVU.MOREX.r3.7HG0665490-RT | TCACATTGATGGTCTGAAGAAAGG | CATCCATCCAAAATGTCCCTGA |
| HORVU.MOREX.r3.7HG0665510-RT | GGCTGGAAGGAGGGAAAGATC | GCCGCTTCTTCTTGGACTTGAC |
| HORVU.MOREX.r3.7HG0665520-RT | CGCCAAAGCCAAGGACCAC | TGTACATGAGGACGAGCTGCC |
| HORVU.MOREX.r3.7HG0665530-RT | GCTTCTGTCACAGGGAACGGA | ACCTGCTCCGTCTCCCGTATCC |
| HORVU.MOREX.r3.7HG0665540-RT | ACACGGTGGAGGTGGTGATGG | TGGTTGGCAACGTACTCGATG |
| HORVU.MOREX.r3.7HG0665560-RT | TGGCAACTGAACGAACGGATG | ACCATTAGGGGGAGGAGTGTCG |
| HORVU.MOREX.r3.7HG0665570-RT | GATGGGTTTGTAGTAGATGGTC | ATGCCACTGCTTGGGTGCTTGC |
| HORVU.MOREX.r3.7HG0665590-RT | CCGCGACACCTTCATATACATG | GTCGCCGGGAAGCATTTCTTG |
| HORVU.MOREX.r3.7HG0665610-RT | AAGTTCAAGATTCAGAGCCACC | GCCGCAACTGCTCAATACAATG |
| HORVU.MOREX.r3.7HG0665620-RT | GCTGAGGAAGTTGCAGGGTAC | ATGACGACGCCGCCGCTATC |
| HORVU.MOREX.r3.7HG0665630-RT | CCGCAGGATAAAGACATTGTTC | GTTATTCTCAAGTATCCTTCCAGCA |
| HORVU.MOREX.r3.7HG0665650-RT | GGAGAAGAATGGGAACTTTGGG | AAGCCCAACCCTTGAAGCCTG |
| HORVU.MOREX.r3.7HG0665680-RT | TCCCTCCTAAGGCGTGACATTG | TCTTGGCGAGCTGAGGGATTTG |
| HORVU.MOREX.r3.7HG0665710-RT | TCCGATTTGATTACTATGCTGC | AGCACAGCTAGAAGCATAAAC |
| HORVU.MOREX.r3.7HG0665730-RT | GAGGAAGGTGCTCGACAGGATC | CCGACACGGTCACCTGGTTC |
| HORVU.MOREX.r3.7HG0665770-RT | TGTTGACCGCTGTGAGGAAG | GGTATCTCCAGCCCAACATCC |
| HORVU.MOREX.r3.7HG0665780-RT | GTCTTTGTGGTCGTCCTCCTGC | GCTGTCTGACCTACACTCCCTC |
| HORVU.MOREX.r3.7HG0665810-RT | TTGCGAGGGATTGTAGGTCAG | TGAAACGCTCGGTCAGGCAAAC |
| HORVU.MOREX.r3.7HG0665830-RT | GAGCGAGAAGGTATTCGTGGAG | CCCTTTGAGAATGGCAGCGTAG |

Table S5 The transcriptions of 23 high confidence annotated genes in the two parents under salt stress

| **The 2-ΔCT value in roots** | | | | | | | | |
| --- | --- | --- | --- | --- | --- | --- | --- | --- |
| Gene ID | 24ck-F | 24ck-T | 24h-F | 24h-T | 48ck-F | 48ck-T | 48h-F | 48h-T |
| HORVU.MOREX.r3.7HG0665410 | 0.0012 | 0.0015 | 0.0008 | 0.0007 | 0.0009 | 0.0012 | 0.0006 | 0.0004 |
| HORVU.MOREX.r3.7HG0665460 | 0 | 0 | 0 | 0 | 0 | 0 | 0 | 0 |
| HORVU.MOREX.r3.7HG0665480 | 0.0036 | 0.0026 | 0.0032 | 0.0023 | 0.0031 | 0.0031 | 0.0056 | 0.0038 |
| HORVU.MOREX.r3.7HG0665490 | 0.0001 | 0.0015 | 0.0002 | 0.0005 | 0.0007 | 0.0003 | 0.001 | 0.0006 |
| HORVU.MOREX.r3.7HG0665510 | 0.0001 | 0.0001 | 0.0001 | 0.0001 | 0 | 0 | 0.0001 | 0 |
| HORVU.MOREX.r3.7HG0665520 | 0.0021 | 0.0025 | 0.0024 | 0.0022 | 0.0022 | 0.0035 | 0.0033 | 0.0029 |
| HORVU.MOREX.r3.7HG0665530 | 0.0817 | 0.1967 | 0.0405 | 0.0565 | 0.1165 | 0.0613 | 0.0786 | 0.0482 |
| HORVU.MOREX.r3.7HG0665540 | 0.0432 | 0.0358 | 0.023 | 0.0219 | 0.038 | 0.0357 | 0.0312 | 0.0202 |
| HORVU.MOREX.r3.7HG0665560 | 0.0002 | 0.0003 | 0.0002 | 0.0001 | 0.0002 | 0.0002 | 0.0001 | 0.0001 |
| HORVU.MOREX.r3.7HG0665570 | 0.0042 | 0.0017 | 0.0051 | 0.0017 | 0.0064 | 0.0014 | 0.0117 | 0.0024 |
| HORVU.MOREX.r3.7HG0665590 | 0.0017 | 0.0038 | 0.001 | 0.0025 | 0.0024 | 0.0052 | 0.0013 | 0.0018 |
| HORVU.MOREX.r3.7HG0665610 | 0.0266 | 0.0402 | 0.0369 | 0.0384 | 0.0391 | 0.0389 | 0.0469 | 0.045 |
| HORVU.MOREX.r3.7HG0665620 | 0 | 0 | 0 | 0 | 0 | 0 | 0 | 0 |
| HORVU.MOREX.r3.7HG0665630 | 0.0067 | 0.0082 | 0.005 | 0.0064 | 0.008 | 0.0087 | 0.0073 | 0.0069 |
| HORVU.MOREX.r3.7HG0665650 | 0.0001 | 0.0001 | 0 | 0.0001 | 0.0001 | 0 | 0.0001 | 0 |
| HORVU.MOREX.r3.7HG0665680 | 0.001 | 0.0011 | 0.001 | 0.0009 | 0.0011 | 0.0015 | 0.0014 | 0.0011 |
| HORVU.MOREX.r3.7HG0665710 | 0.0057 | 0.0057 | 0.0039 | 0.0035 | 0.004 | 0.0055 | 0.0043 | 0.002 |
| HORVU.MOREX.r3.7HG0665730 | 0.0075 | 0.0064 | 0.006 | 0.0048 | 0.0035 | 0.0047 | 0.0042 | 0.0033 |
| HORVU.MOREX.r3.7HG0665750 | 0.0042 | 0.0049 | 0.0148 | 0.0099 | 0.006 | 0.0066 | 0.0148 | 0.0081 |
| HORVU.MOREX.r3.7HG0665770 | 0.0021 | 0.0027 | 0.0024 | 0.002 | 0.0024 | 0.0025 | 0.0031 | 0.0028 |
| HORVU.MOREX.r3.7HG0665780 | 0.0003 | 0.0005 | 0.0005 | 0.0008 | 0.0005 | 0.0006 | 0.0006 | 0.0006 |
| HORVU.MOREX.r3.7HG0665810 | 0.0002 | 0 | 0.0001 | 0 | 0.0001 | 0.0001 | 0.0001 | 0.0001 |
| HORVU.MOREX.r3.7HG0665830 | 0.0013 | 0.0014 | 0.0006 | 0.0006 | 0.0007 | 0.0017 | 0.0008 | 0.0009 |
| **The 2-ΔCT value in leaves** | | | | | | | | |
| Gene ID | 24ck-F | 24ck-T | 24h-F | 24h-T | 48ck-F | 48ck-T | 48h-F | 48h-T |
| HORVU.MOREX.r3.7HG0665410 | 0.0014 | 0.0013 | 0.001 | 0.0008 | 0.0019 | 0.0015 | 0.0025 | 0.0018 |
| HORVU.MOREX.r3.7HG0665460 | 0 | 0 | 0.0001 | 0 | 0.009 | 0 | 0 | 0 |
| HORVU.MOREX.r3.7HG0665480 | 0.003 | 0.0029 | 0.002 | 0.0016 | 0.0046 | 0.0026 | 0.0029 | 0.0029 |
| HORVU.MOREX.r3.7HG0665490 | 0.0003 | 0.0002 | 0.0013 | 0.0004 | 0.0005 | 0.0003 | 0.001 | 0.0003 |
| HORVU.MOREX.r3.7HG0665510 | 0 | 0 | 0 | 0 | 0.0001 | 0 | 0 | 0 |
| HORVU.MOREX.r3.7HG0665520 | 0.0009 | 0.0011 | 0.0011 | 0.0015 | 0.0011 | 0.0013 | 0.0008 | 0.0011 |
| HORVU.MOREX.r3.7HG0665530 | 0.0724 | 0.1003 | 0.0611 | 0.0651 | 0.1637 | 0.1535 | 0.1352 | 0.0778 |
| HORVU.MOREX.r3.7HG0665540 | 0.0362 | 0.0352 | 0.0241 | 0.0206 | 0.0479 | 0.0405 | 0.0343 | 0.0228 |
| HORVU.MOREX.r3.7HG0665560 | 0.0001 | 0.0001 | 0.0001 | 0.0001 | 0.0002 | 0.0002 | 0.0001 | 0.0002 |
| HORVU.MOREX.r3.7HG0665570 | 0.004 | 0.0042 | 0.0053 | 0.0023 | 0.0059 | 0.0007 | 0.0048 | 0.0008 |
| HORVU.MOREX.r3.7HG0665590 | 0.0046 | 0.0063 | 0.0086 | 0.0189 | 0.0096 | 0.0136 | 0.0071 | 0.0204 |
| HORVU.MOREX.r3.7HG0665610 | 0.0223 | 0.0244 | 0.0317 | 0.0285 | 0.0268 | 0.0234 | 0.0224 | 0.0245 |
| HORVU.MOREX.r3.7HG0665620 | 0.0001 | 0 | 0.0001 | 0.0001 | 0.0001 | 0.0001 | 0.0001 | 0.0001 |
| HORVU.MOREX.r3.7HG0665630 | 0.0076 | 0.0116 | 0.0083 | 0.0085 | 0.0102 | 0.0103 | 0.0083 | 0.0091 |
| HORVU.MOREX.r3.7HG0665650 | 0 | 0 | 0.0001 | 0.0001 | 0 | 0 | 0.0001 | 0 |
| HORVU.MOREX.r3.7HG0665680 | 0.0011 | 0.0016 | 0.0012 | 0.0008 | 0.0018 | 0.0015 | 0.001 | 0.0007 |
| HORVU.MOREX.r3.7HG0665710 | 0.0156 | 0.0219 | 0.0043 | 0.0037 | 0.0254 | 0.0199 | 0.0036 | 0.0026 |
| HORVU.MOREX.r3.7HG0665730 | 0.006 | 0.0065 | 0.003 | 0.0038 | 0.0069 | 0.0073 | 0.0033 | 0.0029 |
| HORVU.MOREX.r3.7HG0665750 | 0.0063 | 0.0066 | 0.0182 | 0.0084 | 0.0088 | 0.007 | 0.0759 | 0.0399 |
| HORVU.MOREX.r3.7HG0665770 | 0.0037 | 0.005 | 0.0031 | 0.002 | 0.0044 | 0.0042 | 0.0025 | 0.0017 |
| HORVU.MOREX.r3.7HG0665780 | 0.001 | 0.001 | 0.0006 | 0.0005 | 0.0007 | 0.0008 | 0.0012 | 0.0006 |
| HORVU.MOREX.r3.7HG0665810 | 0.0001 | 0.0001 | 0 | 0 | 0.0001 | 0 | 0.0004 | 0 |
| HORVU.MOREX.r3.7HG0665830 | 0.0081 | 0.0099 | 0.0031 | 0.0019 | 0.0141 | 0.0096 | 0.0025 | 0.0016 |
